# Various vascular malformations are prevalent in Finnish pseudoxanthoma elasticum (PXE) patients: a national registry study

**DOI:** 10.1186/s13023-022-02341-6

**Published:** 2022-05-07

**Authors:** Saku Pelttari, Suvi Väärämäki, Olivier Vanakker, Shana Verschuere, Hannu Uusitalo, Heini Huhtala, Tero Hinkka, Ilkka Pörsti, Pasi I. Nevalainen

**Affiliations:** 1 Faculty of Medicine and Life Sciences, Tampere University, Tampere, Finland; 2 Centre for Vascular Surgery and Interventional Radiology, Tampere University Hospital and Tampere University, Tampere, Finland; 3 Center for Medical Genetics, Ghent University Hospital, Ghent, Belgium; 4 Department of Biomolecular Medicine, Ghent University, Ghent, Belgium; 5 SILK, Department of Ophthalmology, Faculty of Medicine and Health Technology, Tampere University, Tampere, Finland; 6 Tays Eye Centre, Tampere University Hospital, Tampere, Finland; 7 Faculty of Social Sciences, Tampere University, Tampere, Finland; 8 Centre for Vascular Surgery and Interventional Radiology, Tampere University Hospital, Tampere, Finland; 9 Department of Internal Medicine, Tampere University Hospital, Teiskontie 35, 33521 Tampere, Finland

**Keywords:** Finnish-European, Genetics, Pseudoxanthoma elasticum, PXE, Prevalence, ABCC6, Inborn error of metabolism, Registry study, Vascular malformations, Visual acuity

## Abstract

**Background:**

Pseudoxanthoma elasticum (PXE, OMIM# 264800) is an inborn error of metabolism causing ectopic soft tissue calcification due to low plasma pyrophosphate concentration. We aimed to assess the prevalence of PXE in Finland and to characterize the Finnish PXE population. A nationwide registry search was performed to identify patients with ICD-10 code Q82.84. Information was gathered from available medical records which were requisitioned from hospitals and health centers. Misdiagnosed patients and patients with insufficient records were excluded.

**Results:**

The prevalence of PXE in Finland was 1:260,000 with equal sex distribution. Patients with high conventional cardiovascular risk had more visual and vascular complications than patients with low risk. Four patients (19%) had at least one vascular malformation. A high proportion (33%) of *ABCC6* genotypes were of the common homozygous c.3421C > T, p.Arg1141Ter variant. Nine other homozygous or compound heterozygous allelic variants were found.

**Conclusions:**

The prevalence of diagnosed PXE appears to be lower in Finland than in estimates from other countries. Decreased visual acuity is the most prevalent complication. We suggest that various vascular malformations may be an unrecognized feature of PXE.

## Background

Pseudoxanthoma elasticum (PXE) is a rare inherited metabolic disease characterized by increased ectopic calcification of elastic connective tissues. A central finding in patients with PXE is low plasma pyrophosphate (PPi) concentration [1]. Biallelic pathogenic variants in the adenosine triphosphate-binding cassette C6 (*ABCC6*) gene can be found in most patients with PXE. ABCC6 protein (EC 7.6.2.3) regulates adenosine triphosphate efflux from hepatocytes to plasma [2], where ecto-nucleotidase pyrophosphatase/phosphodiesterase 1 (EC 3.6.1.9) converts adenosine triphosphate to PPi and adenosine monophosphate. PPi is one of the foremost substances involved in the inhibition of calcification processes. In aqueous solution, PPi binds spontaneously into polymerization sites in hydroxyapatite crystals in an inhibitory manner, preventing further enucleation and crystallization [3].

Deficiency of PPi predisposes soft tissues to excess calcification. Ocular calcification renders the Bruch’s membrane brittle and prone to fractures that are visualized in ophthalmoscopy as angioid streaks [4]. Cutaneous calcification affects areas of skin folding, appearing as loose and “cobblestone”-like papular or patchy lesions [5]. Vascular calcification in PXE is defined by arteriosclerosis in the medial layer of the small and medium sized arteries, distinguishing it from atherosclerosis of the arterial intima [6]. Increased risk of cerebral and myocardial ischemic events, vascular malformations, gastrointestinal hemorrhage, and kidney stones have been associated with the disease [7–10]. Most symptoms first appear during early adulthood, but dire manifestations have been described even in children [11, 12].

The prevalence estimates of PXE range from 1:25,000 to 1:100,000 with a female predominance of 2:1 [13]. The prevalence of PXE in Finland has not been studied previously. With roughly 5.5 million inhabitants the expected number of patients with PXE in Finland would range from 55 to 220.

## Results

### Prevalence

**Fig. 1:**
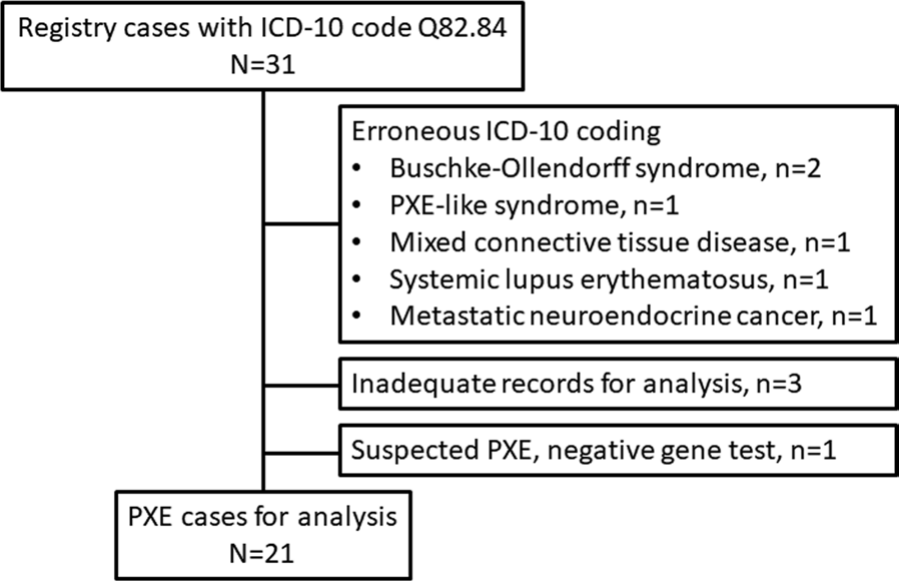
Flow chart

The Finnish population by the end of the year 2018 was 5,517,919 [14]. Registry search produced 31 cases with International Classification of Diseases 10 (ICD-10) code Q82.84 of which ten were excluded (Fig. 1). The diagnosis of PXE was ‘possible’ in two and ‘definite’ in 19 cases (Table 1) [15]. The prevalence of “possible or definite PXE” is thus ~ 1:260,000 while the prevalence of “definite PXE” is ~ 1:290,000 in Finland.

**Table 1:** Patient characteristics

Variables	n/mean	%/range
Patients	21	
Female	11	52.4
Male	10	47.6
Current age (years)	54.7	21–82
Age at diagnosis	33.0	10–63
Diagnostic classification [14]		
Possible	2	9.5
Probable	0	0
Definite	19	90.5
BMI (kg/m^2^)	26.4	19.1–44.6
Family history of PXE	7	33.3
Total cholesterol (mmol/L)	4.9	2.2–6.7
LDL cholesterol (mmol/L)	3.0	1.1–4.8
Hypertension	10	47.6
Type 2 diabetes	3	14.3
Smoking during last 5 years	6	28.6
CVRS		
High risk	8	38.1
Low risk	13	61.9

Reference ranges: BMI 19.0–25.0; total cholesterol < 5.0 mmol/L; LDL cholesterol < 3.0 mmol/L

*PXE* pseudoxanthoma elasticum, *BMI* body-mass index, *CVRS* cardiovascular disease risk score

### Patient characteristics

Patient characteristics are presented in Table 1. Female to male distribution was 1.1:1. Mean age in the study group was 55 (21–82) years. Mean age at diagnosis was 33 (10–63) years. Body mass index (BMI) was available from 16 patients, with a mean of 26.4 (19.1–44.6) kg/m^2^. Seven subjects (33%) were overweight (BMI > 25) and two (10%) were obese (BMI > 30).

Conventional risk factors for cardiovascular diseases were detected in 20 (95%) patients (Table 1). Total cholesterol concentration was 4.9 (2.2–6.7) mmol/L and low-density lipoprotein (LDL) cholesterol 3.0 (1.1–4.8) mmol/L. Six patients (29%) had smoked during the last 5 years. Information about smoking was missing in four (19%) cases. Nearly half of the patients (48%) had hypertension. Three patients (14%) had type 2 diabetes. Cardiovascular risk score (CVRS) was high in eight patients (38%). Mean CVRS score was calculated for both visually impaired (CVRS = 1.8) and non-impaired patients (CVRS = 1.2). Conversely, 4/13 (31%) of the patients with low and 5/8 (63%) of the patients with high CVRS developed visual impairment.

### Ocular characteristics and findings

**Fig. 2:**
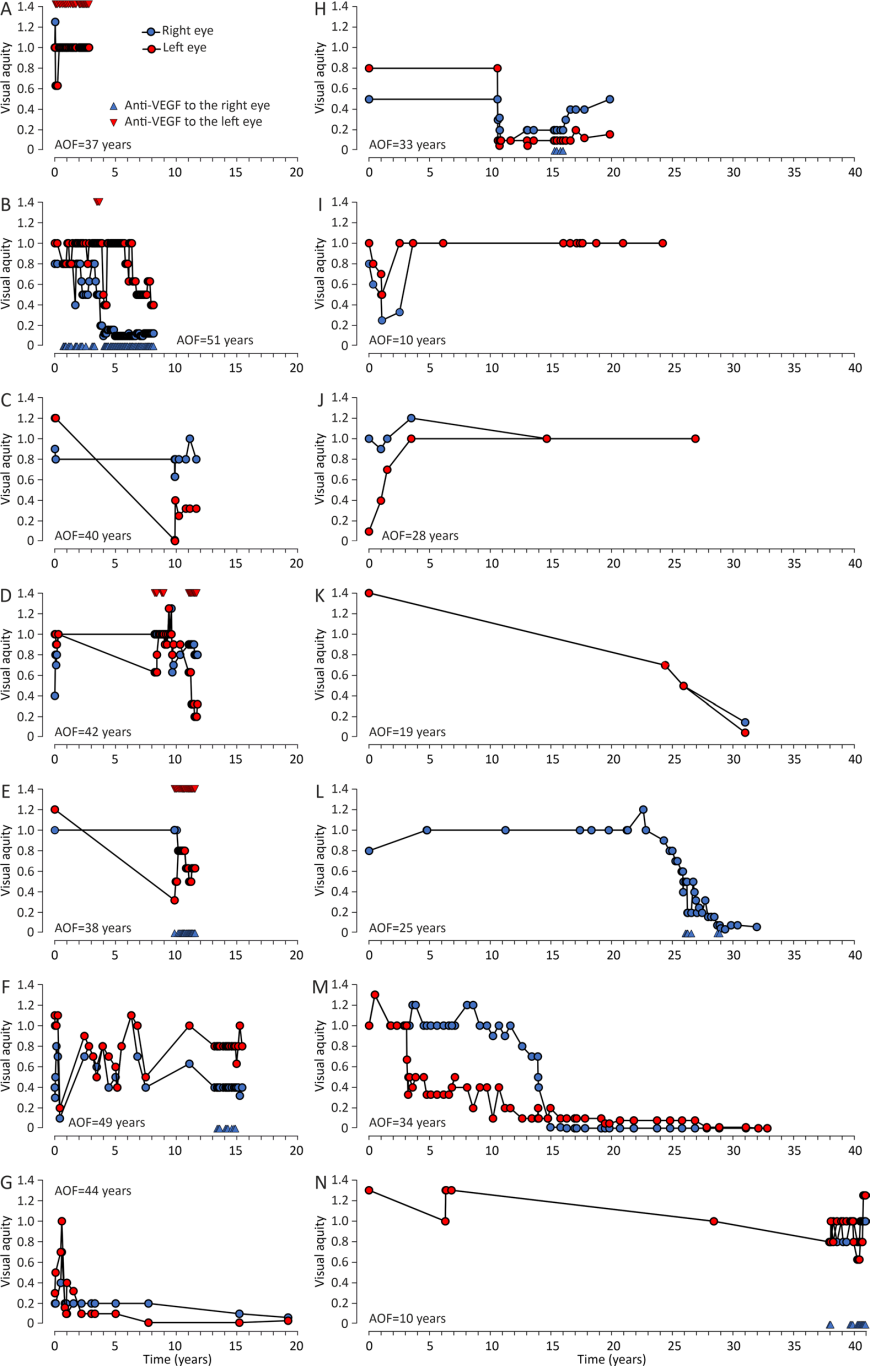
Graphs of best-corrected visual acuity (BCVA) changes during ophthalmological follow-up in 14 individual patients. See panel A for symbol explanations; *AOF* age at the onset of follow-up

Ophthalmological findings are presented in Table 2. Angioid streaks were noted by ophthalmologists in all but one patient, whereas peau d’orange was recorded in 14 patients (67%). Two (9.5%) patients were already legally blind at initial examination. Decrease in best-corrected visual acuity (BCVA) was recorded in 14 patients, of whom four (19%) developed visual impairment during the follow-up. Graphing of the visual follow-up data revealed ten episodes of simultaneous bilateral BCVA decrease (Fig. 2). Mean follow-up was 15.1 (1.7–40.9) years. Mean BCVA was 0.57/0.46 at the first and 0.30/0.24 at the latest ophthalmologist’s appointment. Five patients had no decrease in BCVA during their follow-up median of 5.2 years (1.7–23.1). Excluding visually stable patients, BCVA decreased 0.05/0.05 LogMAR/year on average. BCVA decreased at similar rates between patients who had (0.048 LogMAR/year) and who had not (0.047 LogMAR/year) received intravitreal vascular endothelial growth factor (VEGF) inhibitor injections. In the beginning of the follow-up, one patient had a simultaneous decrease in BCVA and right-sided stroke leading to left hemiplegia (Fig. 2j).

**Table 2:** Ocular characteristics and findings

Variables	n/mean	%/range
Peau d’orange	14	66.7
Angioid streaks	20	95.2
Intravitreal VEGF inhibitor injections administered	12	57.1
Prevalence of visual impairment		
First examination	2	9.5
Latest examination	6	28.6
Mean follow-up (years)	15.1	1.7–40.8
Mean BCVA (right/left)		
First examination	0.57/0.46	0.0–2.0 / 0.0–2.0
Latest examination	0.30/0.24	0.0–1.25/0.0–1.25
Mean BCVA decrease (LogMAR/year)	0.055/0.047	0.018–0.105/0.025–068
Treated	0.048	
Untreated	0.047	

*VEGF* vascular endothelial growth factor, *BCVA* best-corrected visual acuity, *LogMAR* logarithm of minimum angle of resolution

### Cardiovascular diseases and vascular pathologies

Cardiovascular diseases and relevant findings are presented in Table 3. Six patients (29%) had peripheral arterial disease (PAD) confirmed either by magnetic resonance angiography (MRA) or computed tomography angiography (CTA). Ankle-brachial index (ABI) was measured in 8 cases (38%) and five patients had decreased ABI results in both lower limbs. Four of the six patients with claudication were treated conservatively. Two patients (10%) developed critical limb ischemia (age 57 and 59 years) and underwent operative treatment: one distal bypass and one femoral endarterectomy. One patient was asymptomatic despite extensive peripheral artery occlusions.

**Table 3:** Extraocular manifestations and findings

Variable	n/mean	%/range
Skin biopsy	13	69.9
Positive	12	57.1
Cerebrovascular arterial disease	5	23.8
Patients with vascular malformations	4	19.0
Individual malformations	5	
Intra-abdominal hemorrhagic event	6	28.6
Nephrolithiasis	4	19.0
Peripheral artery disease	6	28.6
Claudication	5	23.8
Critical limb ischemia	2	9.5
Ankle-brachial index measured	8	38.1
Abnormal results	5	23.8
Mean abnormal ABI (right/left)	0.64/0.56	0.38–0.77/0.40–0.78

*ABI* ankle-brachial index

**Fig. 3:**
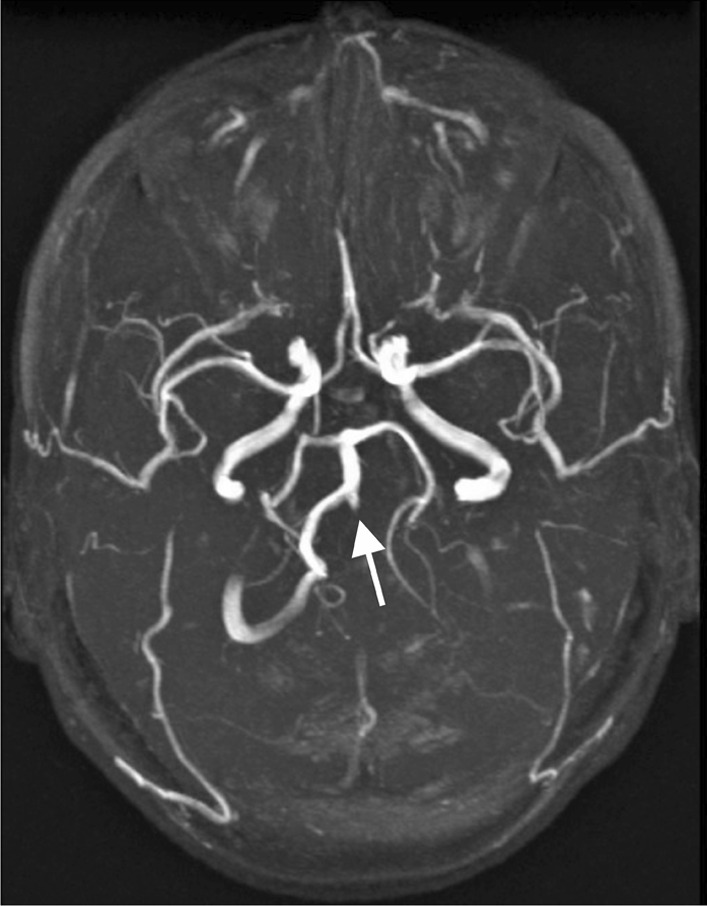
Magnetic resonance angiography of a pseudoxanthoma elasticum patient’s cerebral arteries. The patient had exhibited ischemic symptoms of the vertebrobasilar area in addition to symptoms of right hemisphere ischemia. The left vertebral artery appeared as a mere stump on the left side of the basilar artery and was diagnosed as a subtotal occlusion suspected to be caused by a dissection. Subsequently, a percutaneous intervention was performed

**Fig. 4:**
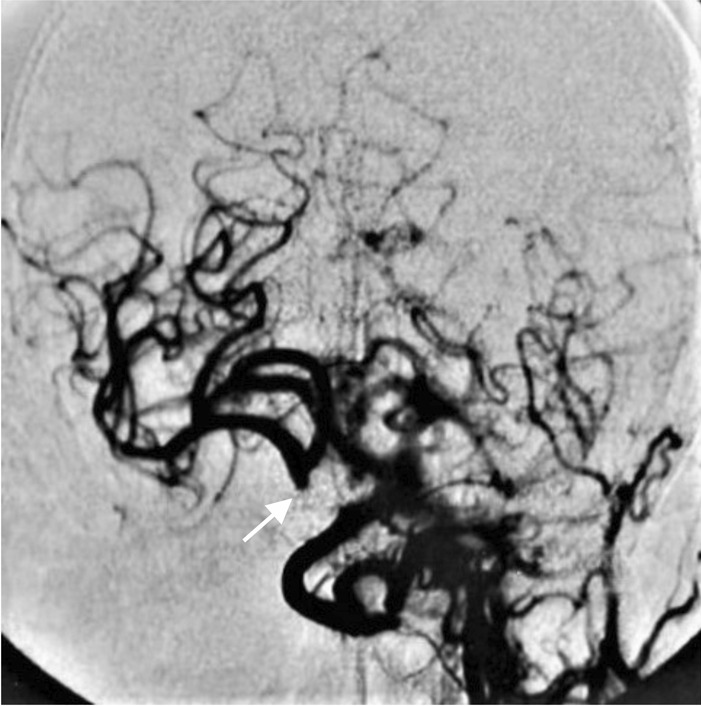
Digital subtraction angiography of a patient’s cerebral arteries. Only a stump of the right internal carotid artery can be visualized (arrow). The patient’s left eye was blind at birth and in a subsequent investigation loss of vision was postulated to have occurred due to a steal effect on the ophthalmic artery, caused by a bilateral carotid artery dissection. The patient was thought to have survived due to extensive anastomosing of carotid and cerebral arteries. The dissected portion of the left carotid artery is not visible. Retrospectively we hypothesize this to be a manifestation of either generalized arterial calcification in infancy type 2, or internal carotid artery hypoplasia

**Fig. 5:**
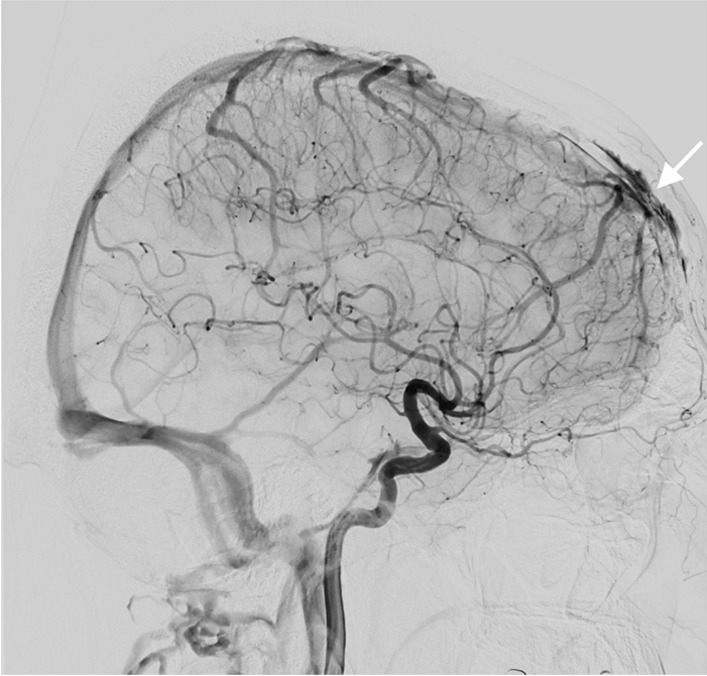
Digital subtraction angiography of cerebral arteries of a patient with pseudoxanthoma elasticum. An aneurysmatic bone cyst of the frontal bone is visualized on the upper left side of the skull (arrow). The patient has had a slight bulging formation on his frontal bone since childhood

Three patients had six strokes (first event at the age of 28, 60 and 61) and one had an intracerebral hemorrhage (age 56 years). Additionally, one patient aged 37 years had a stroke associated with subtotal left vertebral artery dissection (Fig. 3). Another complex craniocervical vascular malformation -associated infarction was confirmed by MRA at the age of 34 years, but was considered to have caused a congenital blindness of the left eye (Fig. 4). In addition, one patient had a lytic lesion of the frontal bone containing a venous aneurysmatic bone cyst, fed by the superior sagittal sinus (Fig. 5). One more patient had a renal artery malformation confirmed by CTA (radiographic images had been disposed of due to expiration), and an endoscopically identified Dieulafoy’s malformation as the culprit lesion in a recurrent gastric hemorrhage. Thus, a total of five vascular malformations were found in four patients (19%). None had any records of coronary artery disease or myocardial infarctions.

PAD and/or cerebrovascular events were found in 75% (6/8) of the patients with high, and in 31% (4/13) with low CVRS.

### Intra-abdominal events

Records of intra-abdominal events are presented in Table 3. A gastrointestinal hemorrhage occurred in six (29%) patients with median age 28 years (9–41), recurrently in one. An emergency laparotomy was performed to a 13-year old patient due to an intra-abdominal hemorrhage originating from an ovarian luteal body. No etiological clues were observed during the operation.

Two (9.5%) cases of radiologically confirmed kidney stones were reported while another two patients had a clinically apparent episode of passing nephrolithiasis (age range 44–51 years).

### Genetics

Biallelic *ABCC6* variants and their pathogenicity are presented in Table 4. The tests were performed on 13 patients (62%). Eight (38%) homozygous and five (24%) compound heterozygous variants were found. Of the homozygous patients, seven (33%) had c.3421C > T, p.Arg1141Ter and one had a homozygous multi-exon intragenic deletion. Additionally, c.3421C > T, p.Arg1141Ter was present in 58% of all *ABCC6* variant combinations found.

**Table 4:** Gene test results and pathogenicity ratings according to ACMG and Sherloc

Patient	*ABCC6* variant allele 1	ACMG	Sherloc	*ABCC6* variant allele 2	ACMG	Sherloc
1	NA			NA		
2	c.3421C > T, p.Arg1141Ter	5	5	c.3421C > T, p.Arg1141Ter	5	5
3	NA			NA		
4	NA			NA		
5	NA			NA		
6	c.3421C > T, p.Arg1141Ter	5	5	c.3421C > T, p.Arg1141Ter	5	5
7	c.1171A > G, p.Arg391Gly	3	3 [16]	Deletion of exons 2–30	3	3
8	c.3421C > T, p.Arg1141Ter	5	5	c.3421C > T, p.Arg1141Ter	5	5
9	c.3421C > T, p.Arg1141Ter	5	5	c.3421C > T, p.Arg1141Ter	5	5
10	c.1132C > T p.Gln378Ter	3	5^a^	c.1898G > T p.Ser633Ile	3	4
11	c.3614_3615del, p.(Ser1205Cysfs*72)	4	5	c.1999del, p.(Ala667Glnfs*21)	4	5
12	c.3421C > T, p.Arg1141Ter	5	5	Partial deletion in exons 2–4	5^b^	3^b^
13	c.341C > G p.Thr114Arg	3	3^c^	Deletion of exon 21	5^b^	3^b^
14	c.3421C > T, p.Arg1141Ter	5	5	c.3421C > T, p.Arg1141Ter	5	5
15	c.3421C > T, p.Arg1141Ter	5	5	c.3421C > T, p.Arg1141Ter	5	5
16	deletion of exons 23–28 c.[2996-? 4041 + ?del]	4	5	deletion of exons 23–28 c.[2996-? 4041 + ?del]	4	5
17	NA			NA		
18	NA			NA		
19	NA			NA		
20	NA			NA		
21	c.3421C > T, p.Arg1141Ter	5	5	c.3421C > T, p.Arg1141Ter	5	5

*ACMG* American College of Medical Genetics, *NA* not available

^a^Pseudogene amplification was excluded

^b^Tendency towards likely pathogenic if the deletion induces a reading frame shift

^c^Pseudogene amplification was not excluded

Nine other allelic variants were found only once in the series. Segregation analysis was performed on only one patient. According to American College of Medical Genetics (ACMG) classification, six variants were “pathogenic” or “likely pathogenic” and four were “variants of unknown significance”. Sherloc classification rated six and five, respectively. The use of Sherloc classification increased pathogenicity rating in 6 and decreased it in 2 alleles. Three single nucleotide variants were in the intracellular (Table 4; patients 7 and 10) and one in the transmembrane (Table 4; patient 13) region. Five variants were exon deletions of varying location and extent. Considering alleles with partial deletions of exons 2–4 and complete deletion of exon 21, the occurrence of a frameshift mutation could not be confirmed, since raw sequence data was not available.

### Other diagnostic procedures

Sixteen patients (76%) had cutaneous lesions associated with PXE. A skin biopsy was taken from 17 (81%) patients. The report was positive for PXE in all but one patient, who had no visible skin lesions.

Confirmed PXE in two cases lead to family investigations in their siblings. One had angioid streaks at the age of ten years and another was confirmed genetically at the age of 51 years. Thirteen patients were initially diagnosed by an ophthalmologist and five by a dermatologist. Information on initial diagnostics was missing in one case.

## Discussion

### Prevalence and patient characteristics

The worldwide prevalence of classical PXE has been estimated to be closer to 1:25,000 than the previously suggested 1:100,000. However, according to heterozygote carrier rate estimations, the prevalence of individuals with biallelic *ABCC6* pathogenic variants could be as high as 1:4450 in France [13]. Remarkably, the Finnish prevalence is less than 10% of that given in contemporary estimates (1:260,000).

It is unknown whether the low prevalence of PXE in Finland is caused by undiagnosed cases or genetic background. Rare diseases are challenging to identify, even when pathognomonic findings are present. The manifestations of PXE are easily confused with more prevalent diseases, such as PAD, age-related macular degeneration, and atherosclerosis. Also, not all biallelic *ABCC6* pathogenic variants associate with the classical PXE-phenotype, which may further complicate the diagnostic process [13].

European prevalence of genetic diseases and variants are separated to Finnish and non-Finnish European genetic pools as explained by “the Finnish Disease Heritage” [17–19]. In the Finnish population, scarcity of PXE may result from the divergent genetic background [20]. This may be reinforced by the notion that our cohort included two “definite PXE” patients of non-Finnish genetic origin. The prevalence of Finnish PXE of “possible or definite PXE” could be as low as 1:320,000.

### Ocular characteristics and findings

Retinal angioid streaks and peau d’orange followed by choroidal neovascularization are hallmarks of PXE [4]. Angioid streaks were found in all but one patient. Reports of peau d’orange lesions were infrequent despite their precedence to angioid streaks in the order of formation. In addition, peau d’orange was sometimes not named, but was reported in a descriptive manner, possibly reflecting challenges in recognition.

The follow-up examinations were mostly executed by ophthalmologists. Thus, visual acuity was among the most frequently reported parameters. Annual mean BCVA decrease was notable, as on average it would take only 9–11 years to progress from normal 1.0/1.0 vision to visual impairment. However, this does not depict BCVA decrease in practice. Follow-up data revealed how BCVA changes were episodic in nature, instead of a steady BCVA decrease over time. The often-bilateral nature of these episodes may suggest a systemic trigger.

There was substantial heterogeneity among patients with PXE regarding the severity of ocular findings and visual outcomes. The variation is most likely affected by bias due to exclusion, as some patients were routinely excluded from BCVA follow-up due to severe visual impaired or legal blindness at the time of diagnosis. Variation could also be explained by several environmental and genetic factors. The known risk factors for age-related macular degeneration like smoking, sun exposure, poor diet, and oxidative stress that bear close resemblance to choroidal neovascularization seen in PXE-patients may have a role [21, 22] Correspondingly, visually impaired patients in this study had more cardiovascular risk factors and a higher CVRS than patients with normal and stable BCVA.

Intravitreal VEGF inhibitor injections appear to have been well integrated into standard PXE treatment regimen as 12/16 patients with choroidal neovascularization had received at least one series of injections. However, the efficacy of intravitreal VEGF injections could not be assessed in this study.

### Association between cardiovascular risk factors and diseases

High prevalence of strokes has been previously reported in PXE populations [23, 24]. Stroke prevalence in our PXE-cohort was 29%, which is more than an order of magnitude higher than the global population prevalence 1.1% [25].

PXE-patients with ischemic cerebrovascular diseases have more cardiovascular risk factors compared with those without cerebral involvement, suggesting an acceleration of soft tissue calcification [24]. Our data supports this notion as the high cardiovascular risk group had 2.5-fold cardiovascular morbidity compared with the low-risk group. There is evidence that pathogenic ABCC6 variants cause not only arteriosclerosis but also atherosclerosis by affecting lipoprotein metabolism [26].

The prevalence of arterial hypertension has been reported to be similar in the Dutch PXE-cohort (20%) and in the population estimates (21%) [24, 27]. Every second patient in our cohort exhibited arterial hypertension, reflecting the population prevalence of arterial hypertension in Finland [28]. Coronary disease is rare in patients with PXE, and accordingly, our cohort exhibited no myocardial infarctions or radiographically established cases of coronary artery disease.

The PAD marker ABI is decreased in 45% of patients with PXE and among those, 95% have associated arterial calcifications [29]. The prevalence of PAD in our study (29%) did not reach that of the previously reported, possibly due to lack of data: ABIs were available for only eight patients. It is also worth noting that while operative treatment is rarely needed and may even be harmful [30], two of our patients developed critical limb ischemia and were operatively treated without further thrombotic events [31]. Despite wide-spread occlusive arterial findings in CTA and MRA in four of the present patients, they suffered only from mild claudication as they continued their walking training.

Considering the association between the cardiovascular morbidity and risks in the Finnish patients with PXE, effective control of common risk factors is very likely to decrease morbidity. Smoking cessation, regular physical exercise, and balanced diet are warranted, even if the evidence of the efficacy of these measures in patients with PXE is not available. In addition, good control of existing arterial hypertension, type 2 diabetes, and hypercholesterolemia appears prudent.

### Vascular malformations

Internal carotid artery hypoplasia (ICAH) has been reported to be extremely rare in the general population, with slightly more than 100 case reports in current literature [32, 33]. It has recently been shown to be considerably more frequent (8.6%) in patients with PXE [9]. In addition, 39% of PXE patients with ICAH have intracranial aneurysms or arterial malformations [9]. Although ICAH was not found in any of our patients, one patient presented with bilateral internal carotid artery occlusion from birth and was thought to have survived due to pre-existing anastomoses of the carotids (Fig. 4). According to previous literature, arterial hypoplasia is easily confused with a dissection in radiographic interpretations [34]. Furthermore, such anastomoses may manifest in conjunction with ICAH [9, 32, 35].

An unprecedented finding in our case series was the propagation of vascular malformations or hemorrhages to six separate vessels or vascular beds: frontal bone (Fig. 5), renal, gastric, ovarian and both carotid and vertebral arteries. Such wide-spread vascular pathology supports the current paradigm of PXE as a systemic disease. Elastic fiber fragmentation is an essential pathogenetic feature of PXE and regulates vascular wall development [36]. Also, fibroblast and endothelial cell dysfunction could relate to vascular wall fragility [9, 37]. More studies are required to explain the disseminated nature of the vascular lesions.

It has been previously suggested that patients with PXE with internal carotid artery hypoplasia should be screened for cerebral aneurysms [9]. We recommend maintaining a low threshold for radiographic evaluation of localized symptoms and findings suggesting vascular abnormalities, as they can lead to dire complications if left untreated.

### Kidney stones

PPi deficiency is known to mediate calcium phosphate crystal deposition in the kidneys [38]. Accordingly, patients with PXE are at a high risk for nephrolithiasis, with a lifetime incidence of 10% and prevalence ranging from 10 to 40% [10, 39]. The nephrolithiasis prevalence of 19% in our cohort supports earlier reports. Of note, the youngest patient with a kidney stone passing was 5 years old at the time of the diagnosis.

### Intra-abdominal hemorrhage

The pathogenesis of gastrointestinal hemorrhage in PXE is not entirely clear, but it has been postulated to be related to gastric artery fragility caused by fragmentation of arterial elastic lamellae [40]. In our study, two hemorrhages manifested during childhood, one of which recurred twice during the later years. Previous literature consists mostly of case reports, but the prevalence of gastrointestinal hemorrhage in PXE populations has been estimated to be 13% [7]. A prevalence of 29% was noted in our cohort, which is more than twice in comparison with the above estimate.

### Genetics

We found no meaningful difference in allele frequency between the Finnish and the non-Finnish populations after performing a comparative prevalence search of the best-known c.3421C > T, p.Arg1141Ter pathogenetic *ABCC6* variant with gnomAD. However, this common pathogenic variant presented with a clear predominance (58%) in our patients when compared with the previously published numbers (25–29%). We found no cases of the other common del23-29 variant [41, 42]. Excluding the c.3421C > T, p.Arg1141Ter variants, the pathogenic variant carrier rate appears lower in Finland in comparison with other ethnic regions.

### Strengths and limitations

The Finnish health care system relies on public hospitals and health care centers, while private health care has a minor role. Public health care records and registries contain comprehensive data of the health of the population, allowing for a good overall image of diagnosed PXE in Finland.

The quality of a registry study considering a rare disease is dependent on the correct ICD-10 code. Any shortcomings in the ICD-10 code entries will inevitably confound the prevalence. Additional inquiries to all university hospitals in Finland suggest that diagnosed cases were not missed. Individual cases may still have been lost, since private health care registries were unavailable to us. On the other hand, there was wide variability in performing diagnostics studies in different health care organizations, which led us to include not only the confirmed but also possible cases.

Medical terminology was somewhat variable in describing retinal findings and BCVA in different hospitals and organizations, hindering the interpretation of the ophthalmologic data. In addition, we had no access to ocular computed tomography data. Skin biopsies were analyzed only by hematoxylin–eosin staining, but characteristic findings were adequately seen in all but one case.

Medical registry data from the past four decades contains confounding factors due to advancement of diagnostic methods and medical reporting. All radiologic reports were available, but some images had been disposed of.

The quality of the genetic data was suboptimal in this register study. The genetic analyses were performed by several different accredited laboratories, while one laboratory used methods distinctly different from the others. Multiplex ligation-dependent probe amplification (MLPA) was performed using a kit assay, which could identify del23-38 but not del23-29, as exon 29 is not included in the primer mix. Thus, del23-28 variants reported in this study could in fact be del23-29. Also, del2-30 raw sequence data was not available. As segregation analysis was performed in only one case, we cannot be sure whether the other heterozygous variants are in trans or cis formation. Pseudogene analysis was not performed in any of the cases.

## Conclusions

The prevalence of diagnosed PXE in Finland appears to less than 10% of that reported in other countries. Uniformly occurring, simultaneous, bilateral episodes of visual acuity decrease suggest an unknown systemic trigger. Vascular pathologies are common especially among patients with other cardiovascular risk-factors. Vascular malformations seem to be an underrecognized complication of PXE. Systematic evaluation of PXE populations in specialized centers are needed to uncover the extent of patients’ clinical manifestations.

## Methods

Our aim was to determine the prevalence of PXE in Finland and to characterize the PXE patient population using a nationwide registry search of the ICD-10 code Q82.84 in the Finnish Care Register for Health Care.

All patient record registries of the Finnish public health care are administrated and regulated by the Ministry of Social Affairs and Health. The registries cover a wide range of medical data in digitized archives. Data is routinely gathered from all age groups and is conditionally available for research purposes. Individual social security numbers enable record linkage across separate registries.

We applied for a list of social security numbers and medical records of the patients with the ICD-10 code Q82.84 from the Care Register for Health Care, a patient data registry overseen by a Ministry of Social Affairs and Health subsidiary, the Finnish Institute for Health and Welfare (THL). Our application was approved (THL/628/5.05.00/2018 and THL/522/5.05.00/2020). Clinical data was acquired from all health care institutions which participated in making the diagnosis, follow-up, and treatment of patients with PXE. Laboratory results and patient records from all specialties were retrieved.

In adherence to the diagnostic criteria devised by Plomp et al. all cases with at least a “possible PXE” were included [15]. Flow chart presents excluded cases (Fig. 1). In the included cases, we specifically searched for information about demographics, ocular manifestations, conventional cardiovascular risk factors, complications of PXE, and *ABCC6* allelic variant data. A cardiovascular risk score (CVRS) of 0–4 points for each patient was devised by summing up the four conventional cardiovascular risk factors (active smoking during last 5 years, LDL cholesterol ≥ 3.0 mmol/L, arterial hypertension, and type 2 diabetes), each contributing one point [21]. Cardiovascular risk was considered to be increased if the score was ≥ 2.

Age at diagnosis was determined from the date when the ICD-10 code for PXE was found for the first time in the patient records, when preceded by a diagnostic finding in ocular examination, skin biopsy and/or genetic testing. The latest available data for BMI, total cholesterol and LDL cholesterol was gathered.

Skin biopsies were regarded valid when taken from a patch of papular or “cobblestone”-like lesion. Biopsy results were deemed positive for PXE if the pathologist had identified fragmented, thickened, aggregated and disorderly elastic fibers in the mid-dermis with evidence of elastic fiber calcification in specialized stains.

PXE-specific lesions and BCVA data, measured either by Snellen or Early Treatment of Diabetic Retinopathy Study charts, were extracted from ophthalmologists’ records. BCVA data is presented in a decimal form and in a right eye/left eye format. Angioid streaks and peau d’orange lesions were deemed present when mentioned or if a series of characteristic retinal changes were described. The date and result of the first and the latest measurement of BCVA by an ophthalmologist was recorded as well as the use of intravitreal VEGF injections.

Mean BCVA in the beginning and at the end of the study period and mean follow-up time were recorded. Follow-up BCVA data of cases showing BCVA changes is presented in Fig. 2. Patients with legal blindness (n = 2), traumatic decrease of BCVA (n = 1) and no BCVA changes (n = 3) were excluded. All calculations of BCVA were performed using the logarithm of minimum angle of resolution (LogMAR) units to enable linear operations and ensure comparability and consistency of reporting. LogMAR-units were transformed into decimals by solving their negative powers of ten. Patients with BCVA below 0.33 (Snellen fraction 6/18 m) in the better eye were classified as visually impaired in accordance with the Finnish national classification of visual impairment. The classification is derived from ICD-10 classification, where BCVA of < 6/18 m corresponds to low vision and blindness.

Presence of hypertension or type 2 diabetes were recorded if the patient had an appropriate ICD-10 code, or a regular prescription of applicable medication that was mentioned in the records. Records of ABI were collected. A diagnosis of PAD was considered if the records presented an abnormal ABI (< 0.9 or > 1.4) or if extensive peripheral vascular calcification was seen in MRA or CTA. Vascular malformations were confirmed and recorded when evidence of such findings emerged in endoscopy or angiography [43].

Gastrointestinal hemorrhage was determined by written records of hematemesis, hematochezia, or melena. Records of other intra-abdominal hemorrhages were included. We also searched for documentation of radiographically confirmed nephrolithiasis and descriptions of clinically apparent episodes of kidney stone passing (colicky back, flank, lower abdominal and/or inguinal pain and macro- or microscopic hematuria).

*ABCC6* variant sequences were analyzed by four different accredited laboratories: ACMG Amsterdam, the Netherlands; Center for Medical Genetics, Ghent, Belgium; Sheffield Diagnostic Genetics Service, United Kingdom; Blueprint Genetics Inc., Finland. In the first three laboratories, nucleotide sequences were analyzed by the Sanger sequencing method, whereas deletions were analyzed by MLPA, using a commercial kit (SALSA MLPA Probemix P092-C1). Pseudogene sequences were excluded by the primers used during Sanger sequencing. As an exception, Blueprint Genetics Inc. used an exome capturing for sequence analysis and copy number variation algorithm for analysis of deletions and duplications.

We classified *ABCC6* variants first according to the established guidelines for variant interpretation by the ACMG, in which modifiers such as “benign” (class 1), “likely benign” (class 2), “uncertain significance” (class 3), “likely pathogenic” (class 4) and “pathogenic” (class 5) were used to stratify the pathogenicity gradient [44]. Additionally, Sherloc classification (version 4.2) was presented, since the ACMG criteria have been suggested to be ambiguous or to lack specificity [45, 46].

Lastly, variables with normal distribution were expressed as mean (range) and variables with skewed distribution as median (range).

## Data Availability

The data supporting the findings of this study are available from the Finnish Institute for Health and Welfare. Restrictions as per Finnish Data Security Act apply to the usage of the data, thus precluding public availability. Data are available from the authors upon reasonable request and with permission of the Finnish Institute for Health and Welfare.

## Abbreviations

ABCC6: ATP-binding cassette subfamily C member 6 gene
ABCC6: ATP-binding cassette subfamily C member 6 protein
ABI: Ankle-brachial index
ATP: Adenosine triphosphate
BCVA: Best-corrected visual acuity
BMI: Body-mass index
CTA: Computed tomography angiography
CVRS: Cardiovascular risk score
ICAH: Internal carotid artery hypoplasia
ICD: International classification of diseases
LDL: Low density lipoprotein
LogMAR: Logarithm of the minimum angle of resolution
MRA: Magnetic resonance angiography
PAD: Peripheral arterial disease
PPi: Pyrophosphate
PXE: Pseudoxanthoma elasticum
THL: Finnish Institute for Health and Welfare
VEGF: Vascular endothelial growth factor
